# Awareness and Perception Towards Implementation of Dispensing Separation in Malaysia: A Cross-Sectional Study

**DOI:** 10.21315/mjms2021.28.1.11

**Published:** 2021-02-24

**Authors:** Norazila Abdul Ghani, Nur ‘Afini Fithriyaani Kamaruddin, Nurul Innany Mokhtar

**Affiliations:** Pharmacy Unit, Hospital Jitra, Kedah, Malaysia

**Keywords:** awareness, perceptions, dispensing separation, pharmacy, delivery of health care

## Abstract

**Background:**

Dispensing separation (DS) is a critical policy change that will reduce medical costs, improve population health and increase the quality of healthcare in Malaysia. This study aims to determine the awareness and perception of the public regarding the DS methods.

**Methods:**

This cross-sectional study uses a pre-validated, self-administered questionnaire, which has been administered to 200 residents in Jitra, Kedah, Malaysia. Descriptive and inferential statistics have been used to analyse the data.

**Results:**

Females formed 64.0% of the participants, with Malay being the dominant ethnic group (*n* = 167, 83.5%); 77.5% of the participants reported they were aware of the role of pharmacists in the healthcare system. However, 35.0% of the participants reported having never heard the term ‘dispensing separation’ in any mainstream media in Malaysia, whereas 73.5% of the participants reported that a pharmacist was more reliable than a physician in providing medicines once the diagnosis had been made and 77.5% of them acknowledged that pharmacists were experts in the field of medication. There was a significant association between the participant’s awareness and the agreement on perceptions toward the implementation of the DS (*P* < 0.05).

**Conclusion:**

Awareness of the implementation of DS among residents in Jitra is still low. However, there is strong evidence of public support and the benefits of DS in Malaysia.

## Introduction

Medication management consists of two different phases, prescribing and dispensing, which are essential (1). The prescribing phase involves the physician, whereas, dispensing of medication is the role of the pharmacist. Dispensing is defined as the provision of medicine. It consists of verifying the validity of a prescription, checking for drug interaction, solving medication-related problems, counseling on the use of medication, documenting the participant’s medication records, and monitoring the medicine therapy (2).

Dispensing separation (DS) is a critical policy change that will reduce medical costs, improve the health of the population, and increase the quality of health care (1, 3, 4). Many countries, particularly in Asia, such as, Japan, Taiwan, South Korea, Indonesia, Philippines and India (1, 3, 5) have gradually started using DS and prescribing roles. Meanwhile, in Malaysia, private physicians are allowed to prescribe and are also given the right to dispense medications from their clinics. To date, in Malaysia, the existence of a DS policy between pharmacists and medical doctors in private community practice is still under argument and discussion. Although pharmacists are professional and highly skilled healthcare workers, who are specialised in medication therapy, regrettably their skills and knowledge are critically under-utilised in Malaysia. Thus, without DS, the community pharmacist is able to only play a subordinate role. The limited chance to dispense prescription medicines has urged the community pharmacist to diversify into supplying health supplements, food, and personal hygiene and beauty products (6). There is a need to transform the nation’s pharmacy practice. Needless to say, the Malaysian healthcare system needs a legislature to implement the separation of the roles of physicians and pharmacists (7).

Several studies have attempted to look at the issue of DS, focusing mainly on knowledge and perception (7, 8, 9). However, awareness of DS among the Malaysian population has not been explored yet.

Therefore, this study aims to determine the awareness and perception among residents in Jitra, Kedah, with regard to DS, and their opinion of DS, if implemented in Malaysia.

## Methods

### Study Design and Settings

This cross-sectional study involved the general population residing in Jitra, Kedah, Malaysia. Jitra is a town in the Kubang Pasu District, which is located in the northern part of Peninsular Malaysia. It is the largest town and administrative centre of the district. The district is also known as the *Education Valley* in Kedah, because there is a concentration of educational institutions in this area. As of 2020, the estimated suburban population in Jitra stands at nearly 63,489 (10).

This study is a quantitative study that includes self-administered questionnaires, which are used as tools to measure the awareness and perception of the study population with regard to DS.

The residents of Jitra were approached by the investigator in public areas, such as shopping malls, retail outlets, recreational parks, and so on. The participants were then briefed on the purpose and nature of the study. After getting their consent, they answered the questionnaire in the presence of a study representative, who could clarify any doubts.

### Study Instrument, Validity and Reliability

After reviewing similar studies, which assessed the public perceptions on DS, (7, 8, 9), a questionnaire containing 15 statements was created. The first section was a demographic tool to collect the socio-demographic information of the respondents. The second section measured the awareness of the participants on the role of the pharmacist and DS, which was adopted and adapted from a study by Hassali et al. (7). The third section involved measuring the perception of the public toward DS, which was adopted from a study by Hassali et al. (7). All statements were developed using the Malay language. Face and content validity were conducted before the pilot study.

This questionnaire was distributed to three local people, who commented on the statements. Their comments were discussed by researchers, and modifications of a few statements were made. To assess the content validity, the questionnaire was subsequently reviewed by four senior pharmacists, who had at least 10 years of working experience.

The questionnaire contained three sections: i) demographic data (6 items); ii) awareness of DS (6 items) and iii) perception of DS (9 items). The study questionnaires were then piloted among 30 participants. The pilot study declared the tool as reliable, with an alpha value of 0.825. Data from the pilot assessment were not included in the final analysis

### Study Participants

The inclusion criteria were Malaysian aged ≥ 18 years old. The participants were asked for consent before completing the self-administered questionnaire. Those respondents who were unable to understand Malay or the English language, and refused to participate, were excluded.

### Sample Size Calculation

Sample size estimation was calculated using the population proportion formula (11). Prior data indicated that the prevalence of support for the future implementation of DS was 3.2% (7) and the population size was 63,489 (10). If the Type I error probability and precision were 5.0% and 2.5%, we would need to study 190 samples. With an additional 10% dropout rate, the sample size was 209 samples. Finally, we approached 215 local people, but only 200 of them agreed to participate in the study.

### Data Analysis

The data were analysed using SPSS version 20. Descriptive statistics were used to determine the frequency, mean and percentage of the data. Descriptive statistics were used to present the demographic information. Chi-square tests were employed (where appropriate) to test the association among the demographic profiles, awareness, and the perception toward DS. A *P-*value of less than 0.05 was considered to be statistically significant.

## Results

### Demographic Data

Approximately 215 local people were conveniently approached, of whom 200 participants consented and completed the questionnaires, which were used for the final data analysis (final response rate 93.02%). Sixty-four percent of the participants were females, with Malay being the dominant ethnic group (*n* = 167, 83.5%) (Table 1).

### Awareness on Implementation of Dispensing Separation in Malaysia

The awareness of participants toward the roles of pharmacists and DS are summarised in Table 2. Sixty-five percent of the participants had never heard of the term ‘dispensing separation,’ in any mainstream media in Malaysia. They did not know that this system had been implemented in other countries. The participants were aware of the pharmacists’ role in dispensing medicines (77.5%). Most participants reported that the pharmacist was more reliable than a physician for providing medicines once the diagnosis had been made, and they also acknowledged that the pharmacists were experts in the field of medications. They also were aware (79.5%) that pharmacists were trained in clinical pharmacy and had learned about diseases, symptoms and treatment. In other words, they accepted pharmacists as being experts in medications. Hence, a majority of them (66.5%) gave their support for future implementation of DS in Malaysia.

### Perception on Implementation of Dispensing Separation in Malaysia

The results in Table 3 indicate that most participants were in agreement with the perception of implementing DS in Malaysia. Among all the items explored, the participants gave a higher percentage (76.5%) for optimisation of medication safety, wherein, they felt safer if the prescription prescribed by their doctor was screened through and double checked by the pharmacist.

There were some participants (26.5%), who were concerned whether the implementation of DS would cause inconvenience to the consumer, in getting the medication. This study also found that 66.5% of the respondents expressed their support for the separation policy to be implemented in Malaysia, whereas 29.5% of the respondents gave a neutral agreement.

### Relationship between Demographic Variables, Awareness and Perception of Implementation of Dispensing Separation in Malaysia

Most of the demographic variables were not significantly associated with the perception of implementation of DS (Table 4). Gender, educational background and monthly income were the only demographic variables significantly associated with one of the perception variables (*P* < 0.05).

The association between the awareness and perceptions of respondents toward the implementation of DS are also summarised in Table 5. The results showed a strong association between the participants’ awareness and their agreement on perceptions toward the implementation of DS (*P* < 0.05). Almost all the items under perception significantly correlated with the awareness of DS.

## Discussion

### Awareness of Implementation of Dispensing Separation in Malaysia

Diagnosis and dispensing are two processes that should be handled by two different healthcare professionals for an effective system of checks and balances. This helps to reduce medication error and removes the preference of certain products or overprescribing in the presence of monetary enticement, thereby, promoting rational drug use based on the clinical guidelines (12).

In Malaysia, the healthcare system is a two-tier system comprising of public and private sectors. DS has been implemented in government healthcare facilities for decades. However, this is not being practiced in the private sector (12). This study aims to draw the attention of the public on this issue.

The awareness of the implementation of DS among residents in Jitra, Kedah, is still low. This may be due to lack of campaigning activities for promoting the role of pharmacists and the awareness of DS in this area. Most of the participants accept pharmacists as medicine experts and a majority of them have given their support for future implementation of DS in Malaysia. This finding is in line with what has been reported earlier (7, 8, 9).

### Perception on Implementation of Dispensing Separation in Malaysia

Most participants were positive and agreed on the perception of implementation of DS in Malaysia. Among all the items explored, the participants gave a higher percentage for optimisation of medication safety, which they felt was safer, as the prescription prescribed by their doctor was screened and double checked by the pharmacist. DS was a system that had proved to be beneficial for patients, in terms of improving patient safety and reducing prescribing errors (13). This finding corroborated with the findings of a previous study (9). In the United Kingdom, the rate of prescribing error was reported to be 8.8 (95% confidence interval (CI): 8.6–9.1) per 100 ordered medications (14). The local data showed that the prescribing error was responsible for more than three-quarters (76.1%) of the overall medication error reports (17,357 reports from 97 public hospitals, 117 public clinics, five private hospitals and two teaching hospitals) (15). Thus, DS could help in reducing the incidence of prescribing errors, especially in the private sector. With this well-balanced system, the pharmacist would serve as the last line of defense to identify the prescribing errors and stop them from reaching patients.

Some participants are concerned about the implementation of DS; if it will inconvenience the consumer in getting the medication. In Malaysia, the DS policy has not yet been implemented, possibly due to a historical shortage of pharmacists in this country, which is still in its early stages of development following its independence in 1957. As the country progressed, over the following decades, the number of pharmacists has increased steadily. In 2017, the WHO-recommended pharmacist to population ratio of 1:2000 has been reached (12, 16). Technically, based on this ratio, the country is ready for the separation policy.

More than half of the participants supported the opinion that a separation policy should be implemented in Malaysia. However, there were some respondents who gave a neutral agreement (29.5%). This might be due to the lack of knowledge and understanding of the policy itself. A previous study showed that a range of 52.5% to 87.6% of the respondents supported the future implementation of a separation policy (7, 9). This study has provided strong evidence that the public would support DS in Malaysia if they truly understood the policy and were aware of it.

### Relationship between Demographic Variables, Awareness and Perceptions on Implementation of Dispensing Separation in Malaysia

Gender, educational background, and monthly income were the only demographic variables that were significantly associated with perception variables. During subgroup analysis, the following groups were found to be more likely to agree that a separation policy should be implemented: females, Malay and respondents with a tertiary education. Our findings were consistent with results from other studies (7). These results indicated that these groups of patients were more open and ready to accept new ideas and theories.

There is a strong association between participant awareness and the agreement on perception toward implementation of DS. Lack of public knowledge is perceived as one of the main barriers to a policy shift (9). The Malaysian Pharmaceutical Society (MPS) has led several campaigns in raising consumer awareness on pharmacy services, the role of pharmacists, prescription education (complete prescriptions, patients’ rights to a prescription, the importance of a prescription) and the awareness of DS, with the manifesto of ‘Better and Safer Medicine for the Healthcare of the Rakyat’ (17, 18). However, the campaigns are limited to written information (leaflets, brochures and posters) focusing on urban areas and not across the country. Thus, this is a barrier to the effectiveness of the campaigns.

As an effort to improve the awareness, we recommend MPS to cooperate with the Ministry of Health Malaysia to organise a nationwide campaign in order to enlighten the public on the benefits of DS. The campaign must try various methods, because it is much more effective than concentrating on a single method. It must be done using video, printed material, educational games and a face-to-face approach through digital marketing campaigns (websites, social media), radio and television campaigns, outside-the-box campaigns (e.g. viral video) and the like.

In addition, the roles of pharmacists in the healthcare service, particularly in prescription checking and medication counseling should be emphasised upon, to provide better and safer medicine and improve the public’s perception of pharmacy services.

## Conclusion

Awareness of the implementation of DS among the residents of Jitra, Kedah, is still low. However, there is a strong evidence of public support with respect to the benefits of DS in Malaysia. Further research may be warranted to collect more data, to provide a holistic insight into the general Malaysian’s perception and awareness of DS. These findings are of high relevance to the policy makers, as they provide an overview of the public’s choice of implementing DS in Malaysia.

## Limitation

This study was designed as a self-reported questionnaire. As such they may have systematic biases, particularly in behaviour. Second, the sampling method is convenient, which also gives some bias to be representative of the population being studied. This study was conducted in Jitra, a small town in Northern Kedah, Malaysia. Thus, suburban residents may face health challenges for care, related to geographic barriers, physician shortage, poverty and lower educational background, which may affect their awareness and perception, and thus limit our ability to generalise the study results.

## Figures and Tables

**Table 1:** Demographic characteristics of the study participants

Characteristic		Frequency, *n* (*N* = 200)	Percentage (%)
Age (31.24 ± 11.53 years)	15–24	67	33.5
	25–34	73	36.5
	35–44	31	15.5
	45–54	19	9.5
	> 55	10	5.0
Gender	Male	73	36.5
	Female	127	63.5
Ethnicity	Malay	167	83.5
	Chinese	21	10.5
	Indian	8	4.0
	Others	4	2.0
Highest education level	Primary school	3	1.5
	Secondary school	55	27.5
	Diploma/Degree	135	67.5
	Postgraduate	7	3.5
Monthly income (RM)*	< 2,000	94	47.0
	2,001–3,999	44	22.0
	4,000–5,999	16	8.0
	6,000–8,000	5	2.5
	> 8,000	2	1.0

Note:

* Missing data = 39

**Table 2:** Public’s awareness on implementation of DS in Malaysia

Item in questionnaire		Yes *n* (%)	No *n* (%)
A1:	Have you ever heard the term ‘dispensing separation’ in any mainstream media in Malaysia?	70 (35.0)	130 (65.0)
A2:	Are you aware the role of pharmacist in healthcare system?	155 (77.5)	45 (22.5)
A3:	Do you think that pharmacist is more reliable than a physician in providing medicines upon diagnosis has been made?	147 (73.5)	53 (26.5)
A4:	Do you acknowledge those pharmacists are expertise in the medication field?	171 (85.5)	29 (14.5)
A5:	Are you aware that in some country, pharmacist is eligible in prescribing the medication for patient?	119 (59.5)	81 (40.5)
A6:	Are you aware that pharmacists are trained in clinical pharmacy where they also learn about diseases, symptoms and treatment?	159 (79.5)	41 (20.5)

**Table 3:** Public’s perception on implementation of DS in Malaysia

Item in questionnaire		SD^ϒ^ *n* (%)	D^ϒ^ *n* (%)	N^ϒ^ *n* (%)	A^ϒ^ *n* (%)	SA^ϒ^ *n* (%)
P1:	Implementation of DS will help to reduce medication error.	5 (2.5)	6 (3.0)	73(36.5)	75 (37.5)	41 (20.5)
P2:	Implementation of DS cause consumer’s inconvenience in getting medication.	25 (12.5)	42 (21.0)	80 (40.0)	37 (18.5)	16 8.0)
P3:	Community pharmacist will provide better advice on the use of medicine rather than the staff in private general practitioner’s clinic.	3 (1.5)	8 (4.0)	64 (32.0)	70 (35.0)	55 (27.5)
P4:	Private clinics often overcharge patient by giving unnecessary medicine.	7 (3.5)	18 (9.0)	72 (36.0)	58 (29.0)	45 (22.5)
P5:	Implementation of DS will help to reduce the cost of medication.	4 (2.0)	(6.0)	94 (47.0)	67 (33.5)	23 (11.5)
P6:	I feel safer if my prescription prescribed by my doctor is screened through and double checked by the pharmacist.	2 (1.0)	4 (2.0)	41 (20.5)	82 (41.0)	71 (35.5)
P7:	In my opinion, pharmacists are better position in dispense medication rather than a general practitioner.	4 (2.0)	10 (5.0)	71 (35.5)	60 (30.0)	55 (27.5)
P8:	In my opinion, implementation of DS will optimise patient medication safety.	3 (1.5)	6 (3.0)	61 (30.5)	85 (42.5)	45 (22.5)
P9:	I will support future implementation of DS in Malaysia.	5 (2.5)	3 (1.5)	59 (29.5)	77 (38.5)	56 (28.0)

Notes: SD^ϒ^ = strongly disagree; D^ϒ^ = disagree; N^ϒ^ = neutral; A^ϒ^ = agree; SA^ϒ^ = strongly agree

**Table 4:** Association and relationship between demographic variables and public’s perceptions on implementation of DS

Item in questionnaire		Age	Gender	Ethnic	Educational level	Occupation	Monthly income
P1:	Implementation of DS will help to reduce medication error.	0.385c	0.288^b^	0.288^b^	0.548^b^	0.710a	0.534^b^
P2:	Implementation of DS cause consumer’s inconvenience in getting medication.	0.616c	0.101a	0.097a	0.441^b^	0.233a	0.632^b^
P3:	Community pharmacist will provide better advice on the use of medicine rather than the staff in private general practitioner’s clinic.	0.862c	0.006^b^	0.006^b^	0.934^b^	0.340a	0.477^b^
P4:	Private clinics often overcharge patient by giving unnecessary medicine.	0.819c	0.292^b^	0.292^b^	0.148^b^	0.694a	0.065a
P5:	Implementation of DS will help to reduce the cost of medication.	0.296c	0.232^b^	0.232^b^	0.897^b^	0.184a	0.035^b^
P6:	I feel safer if my prescription prescribed by my doctor is screened through and double checked by the pharmacist.	0.865a	0.400^b^	0.400^b^	0.406^b^	0.795a	0.452^b^
P7:	In my opinion, pharmacists are better position in dispense medication rather than a general practitioner.	0.260c	0.624^b^	0.624^b^	0.034^b^	0.064a	0.469^b^
P8:	In my opinion, implementation of DS will optimise patient medication safety.	0.730c	0.362^b^	0.362^b^	0.898^b^	0.362a	0.040^b^
P9:	I will support future implementation of DS in Malaysia.	0.774c	0.951^b^	0.951^b^	0.494^b^	0.592a	0.925^b^

Notes:

a Pearson’s Chi-square test;

b Fisher’s exact test;

c One-way ANOVA;

*P* < 0.05 is considered significant

**Table 5:** Association between awareness and perceptions towards the implementation of DS

Item in questionnaire		A1: Have you ever heard the term ‘dispensing separation’ in any mainstream media in Malaysia?	A2: Are you aware the role of pharmacist in healthcare system?	A3: Do you think that pharmacist is more reliable than a physician **in providing medicines** upon diagnosis has been made?	A4: Are you aware that in some country, pharmacist is eligible in prescribing the medication for patient?	A5: Do you acknowledge those pharmacists are expertise in the medication field?
P1:	Implementation of DS will help to reduce medication error.	0.297**	0.001**	< 0.001**	0.025**	0.002**
P2:	Implementation of DS cause consumer’s inconvenience in getting medication.	0.168*	0.010*	0.033*	0.075*	0.002*
P3:	Community pharmacist will provide better advice on the use of medicine rather than the staff in private general practitioner’s clinic.	0.116**	<0.001**	<0.001**	0.056**	<0.001**
P4:	Private clinics often overcharge patient by giving unnecessary medicine.	0.159*	0.001**	0.386**	0.008*	0.002*
P5:	Implementation of DS will help to reduce the cost of medication.	0.029**	0.008**	0.030**	0.008**	0.018**
P6:	I feel safer if my prescription prescribed by my doctor is screened through and double checked by the pharmacist.	0.411**	< 0.001**	< 0.001**	< 0.001**	< 0.001**
P7:	In my opinion, pharmacists are better position in dispense medication rather than a general practitioner.	0.114**	< 0.001**	< 0.001**	0.408**	< 0.001**
P8:	In my opinion, implementation of DS will optimise patient medication safety.	0.001**	< 0.001**	< 0.001**	0.005**	< 0.001**
P9:	I will support future implementation of DS in Malaysia.	0.025**	< 0.001**	< 0.001**	0.002**	< 0.001**

Notes:

* Pearson’s Chi-square test;

** Fisher’s exact test; *P* < 0.05 is considered significant
